# Institutional Violence Perpetrated against Transgender Individuals in Health Services: A Systematic Review of Qualitative Studies

**DOI:** 10.3390/ijerph21081106

**Published:** 2024-08-21

**Authors:** Gilberto da Cruz Leal, José Nildo de Barros Silva Júnior, Quezia Rosa Ferreira, Jaqueline Garcia de Almeida Ballestero, Pedro Fredemir Palha

**Affiliations:** Ribeirão Preto College of Nursing, University of São Paulo, Ribeirão Preto 14040-902, Brazil; jose.nildo@usp.br (J.N.d.B.S.J.); quezia@usp.br (Q.R.F.); jaqueline.almeida@usp.br (J.G.d.A.B.); palha@eerp.usp.br (P.F.P.)

**Keywords:** transgender persons, violence, health services, review

## Abstract

This review aims to analyze the evidence related to violence perpetrated against transgender individuals in health services based on their narratives. This is a systematic literature review of qualitative studies. A search was carried out in the Scopus, Web of Science, Latin American and Caribbean Literature in Health Sciences (LILACS), Cumulative Index to Nursing and Allied Health Literature (CINAHL), EMBASE, and MEDLINE databases using the descriptors “transgender people”, “violence”, and “health services”. The eligibility criteria included original qualitative articles addressing the research question, with fully available text, reporting violence specifically by health workers, involving trans individuals aged 18 and above, and published in Portuguese, English, or Spanish. In addition, studies were included that reported experiences of violence suffered by the trans population, through their narratives, in health services. A total of 3477 studies were found, of which 25 were included for analysis. The results highlighted situations such as refusal of service; resistance to the use of social names and pronouns; barriers to accessing health services; discrimination and stigma; insensitivity of health workers; lack of specialized care and professional preparedness; and a system focused on binarism. The analysis of the studies listed in this review highlights the multiple facets of institutional violence faced by the transgender population in health services. It is evident that the forms of violence often interlink and reinforce each other, creating a hostile environment for the transgender population in health services. Thus, there is an urgent need to create strategies that ensure access to dignified and respectful care for all individuals, regardless of their gender identity.

## 1. Introduction

Transgender (trans) is an umbrella term used to refer to transsexuals, transvestites, non-binary, and other possibilities, who do not identify with the binary gender classification (male or female) and heterocisnormativity (which regards identities, genders, and bodies as products regulated by a given society), both associated with the biological anatomical sex at birth (male, female, or intersex) [1,2,3].

Due to their “deviant” behavior and unique characteristics of gender expression, which break away from behaviors marked as “standard”, the trans population is highly marginalized in various spaces [2,3,4], resulting in their exclusion and social erasure. Consequently, they are exposed to stressors, mainly involving family and community violence, which increases stress, anxiety, depressive symptoms, post-traumatic disorders, substance abuse, and suicide cases [4,5,6].

It is known that trans people are frequently exposed to verbal, psychological, and physical violence, and this situation is considered natural in the collective imagination. For these reasons, institutional violence can be observed in various spaces, including health services [7].

Institutional violence can be seen as a set of deleterious situations practiced in service-providing institutions by agents who should meet the demands and protect users but fail to do so, either by omission or commission [8]. It is, therefore, a set of systematic abuses or violations committed by institutions against people or groups, based on power relations and motivated by inequalities involving socioeconomic conditions, ethnicity, age, religion, and gender, among others [9].

The literature considers that institutional violence arises individually, therewith, from the person who practices it. The consistency and high distribution of episodes characterize it as institutional [10].

In health services, the trans population tends to face hostility involving denial of care [11,12,13], verbal harassment, stigma, discrimination [14,15,16,17,18], denial of equal treatment [16,18,19,20], and non-acceptance of the use of social names and appropriate pronouns by staff members [16,17,21,22,23,24].

Despite the significant advances through demands and constant struggles (strikes, marches, lawsuits, court decisions/rulings) achieved by the Lesbian, Gay, Bisexual, Transgender, Intersex, Queer/Questioning, Asexual/Aromantic, Pansexual, Non-binary, and others (LGBTQIAPN+) population worldwide, the transgender population still faces alarming peculiarities. The extremely reduced life expectancy (about 35 years) and episodes of murders motivated by transphobia represent a serious global public health issue [25,26].

Thus, it is assumed that disparities in access to health services directly impact the health conditions of the trans population. This situation can influence the quality (in this case, the lack thereof) and life expectancy of the trans population [17], being considered a grave form of violence against this population. This study aims to analyze the evidence related to institutional violence perpetrated against transgender individuals in health services based on their narratives.

## 2. Materials and Methods

The data collection started after the construction, registration, and publication of the protocol in the International Prospective Register of Systematic Reviews (PROSPERO). The registration number is CRD42024524059.

This is a systematic review of qualitative studies based on the Joanna Briggs Institute (JBI) [27] manual for evidence synthesis of systematic reviews of qualitative research and aligned with the Preferred Reporting Items for Systematic Reviews and Meta-Analyses (PRISMA) [28].

Qualitative research is understood from a complex and interconnected family of terms, concepts, and hypotheses [29]. This type of study aims to explore a phenomenon of investigation (in this case, institutional violence suffered by the trans population in health services) without focusing on statistical quantification and generalization [30]. Moreover, it allows for a more refined understanding of a phenomenon based on the perceptions of subjects, considering their most subjective aspects.

The following steps were defined for this study: identifying the theme and guiding question, establishing inclusion and exclusion criteria, defining the information to be extracted from the selected studies, evaluating the studies included in the systematic review, interpreting results, and synthesizing knowledge.

The research question was defined based on the PICo strategy, where P is the population (transgender persons); I is the phenomenon of interest (violence); and Co is the context (health service). The following question was formulated: What is the scientific evidence related to institutional violence perpetrated against transgender individuals in health services?

The eligibility criteria included original qualitative articles addressing the research question, with full-text availability, reporting violence specifically by health workers, involving trans individuals aged 18 and above, and published in Portuguese, English, or Spanish. In addition, studies were included that reported experiences of violence suffered by the trans population, through their narratives, in health services. Duplicated studies, gray literature texts, and those involving populations and settings divergent from the established eligibility criteria were excluded. Studies that discussed violence against the broader LGBTQIAPN+ population without specific details on the violence suffered by the trans population and their particularities were also excluded.

The term “health workers” was used to encompass individuals holding various positions, including health professionals and other staff, regardless of hierarchical and educational levels [31].

The search for articles was conducted in March 2024 using the DeCS/MeSH descriptors “transgender persons”, “violence”, and “health services” in their respective word banks. Additionally, these descriptors were submitted to PubMed and Web of Science databases to expand the number of synonyms. Based on the synonyms, titles, abstracts, and descriptors of the found articles, the terms for this study were defined (Table 1).

Based on these defined descriptors, a new search was conducted in the Scopus, Web of Science, Latin American and Caribbean Literature in Health Sciences (LILACS), Cumulative Index to Nursing and Allied Health Literature (CINAHL), EMBASE, and MEDLINE databases using the Boolean operators AND and OR, adapted to each database according to their specificities (Table 2). The search strategy was created to be as broad as possible, reducing the possibility of missing relevant articles for this review. It should be noted that all the searches were carried out on 20 March 2024.

The data, after being gathered, were exported to the Rayyan QCRI platform. This platform assists in the process of excluding duplicate studies, as well as in the subsequent reading of titles and abstracts. This first reading was carried out by two independent researchers. A third evaluator was invited to help make decisions (inclusion or exclusion) in cases of disagreement or doubt between the first two. Thus, the studies selected at this stage were subjected to full-text reading, allowing for an analysis of their relevance concerning their inclusion in the review.

Data extraction was carried out using an adapted version of the Lockwood et al. form [32], which included the following variables: author; year and journal of publication; phenomenon of interest (objective); method (study location, participants, data analysis); and main results. The critical reading process was carried out using JBI’s [27] critical evaluation tools. The methodological quality assessment of the studies included in this review was carried out using a checklist for qualitative research evaluation proposed by JBI [27].

## 3. Results

**Figure 1:**
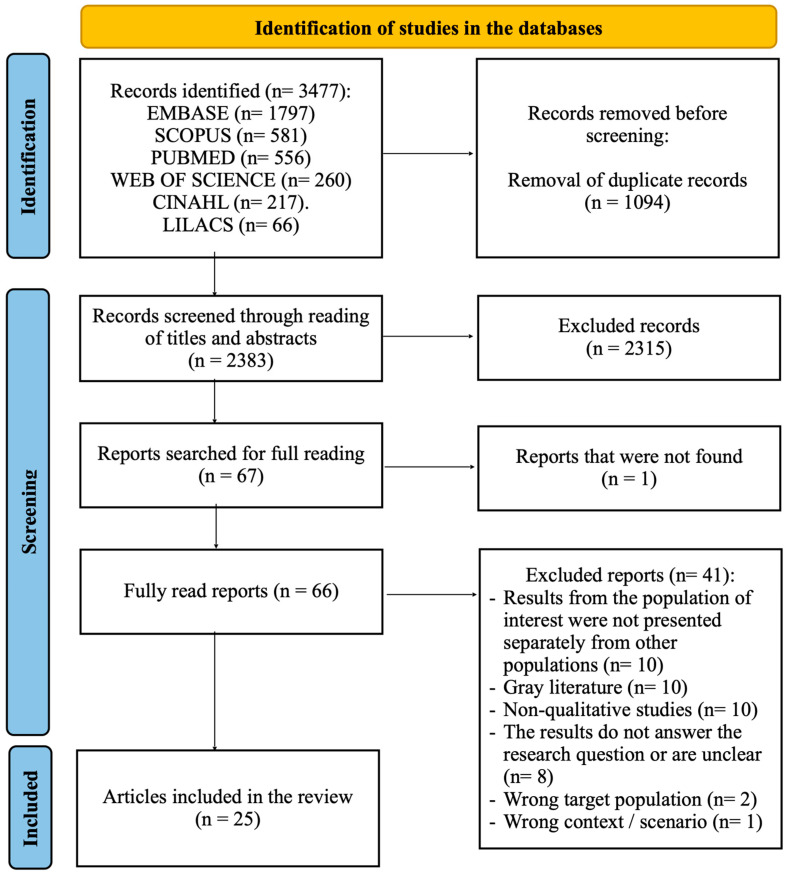
PRISMA flowchart. Source: created from the model by Page et al. [28].

The detailed selection of studies can be viewed in the flowchart (Figure 1). A total of 3477 studies were found in the databases. Initially, 1094 were excluded for being duplicate texts. Of the remaining 2383, 2315 were excluded after reading the title and abstract. Then, the remaining texts were read in full (67 studies, as one was not available). Finally, 42 were rejected after a thorough analysis based on the other eligibility criteria. Thus, 25 [33,34,35,36,37,38,39,40,41,42,43,44,45,46,47,48,49,50,51,52,53,54,55,56,57] studies were used to compose this review (Table 3).

The publication of these 25 articles occurred between 2013 and 2024, with two in 2013 [33,34], two in 2015 [35,36], one in 2016 [37], two in 2018 [38,39], four in 2019 [40,41,42,43], three in 2020 [44,45,46], one in 2021 [47], five in 2022 [48,49,50,51,52], four in 2023 [53,54,55,56], and one in 2024 [57].

Regarding the place of publication, the articles come from 10 countries: Brazil (6 studies) [36,40,45,48,53,54], United States of America (5 studies) [33,34,39,42,52] Canada (4 studies) [35,37,46,56], Uganda (3 studies) [41,51,55], Colombia (2 studies) [38,44], Peru (1 study) [47], Spain (1 study) [49], Nigeria (1 study) [50], India (1 study) [43], and Turkey (1 study) [57].

Concerning the methodological quality of the studies (Table 4), all showed congruence between the research methodology and the research question or objective [33,34,35,36,37,38,39,40,41,42,43,44,45,46,47,48,49,50,51,52,53,54,55,56,57], between the research methodology and the methods used to collect the data [33,34,35,36,37,38,39,40,41,42,43,44,45,46,47,48,49,50,51,52,53,54,55,56,57], between the research methodology and the representation and analysis of the data [33,34,35,36,37,38,39,40,41,42,43,44,45,46,47,48,49,50,51,52,53,54,55,56,57], as well as between the research methodology and the interpretation of the results [33,34,35,36,37,38,39,40,41,42,43,44,45,46,47,48,49,50,51,52,53,54,55,56,57]. Similarly, all studies adequately represented the participants and their voices [33,34,35,36,37,38,39,40,41,42,43,44,45,46,47,48,49,50,51,52,53,54,55,56,57] and brought conclusions aligned with the data collection and analysis [33,34,35,36,37,38,39,40,41,42,43,44,45,46,47,48,49,50,51,52,53,54,55,56,57].

The ethical aspects were clear, evident, and appropriate in 24 studies [33,34,35,36,37,38,39,40,41,42,43,44,45,46,47,48,49,50,51,52,53,54,55,56]. Thus, only one study did not clarify whether it was submitted to a Research Ethics Committee or a similar body in the location where it was conducted [57]. Regarding the statement that locates the researcher culturally or theoretically in the study, 16 articles evidenced that there was this location [33,35,38,40,41,42,43,44,45,46,49,50,52,53,56,57], 8 did not make this aspect clear [34,36,39,47,48,51,54,55], and 1 did not show it [37]. The influence of the researcher was perceived in 8 texts [35,42,43,44,45,46,56,57]. In the others 17 texts [33,34,36,37,38,39,40,41,47,48,49,50,51,52,53,54,55], it was not clear whether there was this type of influence or not.

## 4. Discussion

The violence suffered by the trans population proved to be substantially diversified. Thus, discussion blocks were created based on the participants’ experiences and the authors’ analysis of the studies. This way, it was possible to identify seven blocks, namely: 1. refusal of care; 2. resistance to the use of social name and pronoun; 3. barriers to accessing health services; 4. discrimination and stigma; 5. insensitivity of health workers; 6. lack of specialized care and professional unpreparedness; and 7. technological limitations relating to the binary-focused system. It is worth noting that although the blocks are presented separately, the forms of violence coexist and are interconnected.

Although all the factors represent a potential barrier to accessing health services, we chose to create blocks based on the information that emerged from the studies included in this review. We know that various situations of violence can result in a barrier to access. However, unfortunately, many trans people have to ignore the way they are treated in order to receive the minimum health care.

### 4.1. Refusal of Care

Refusal of care was an evident phenomenon in five studies [34,41,47,51,55]. According to the target population’s reports, the motivations for this refusal were strongly related to their gender identity [47,51] and their way of dressing [41]. In the latter case, a trans woman noted that the professional did not provide quality care if she was wearing a dress. The omission of health workers in providing care was also observed in three studies [34,42,55].

In the study by Kosenko et al. [34], a trans person reported being asked to leave the health facility by the professional who was providing care after expressing a desire to undergo the gender affirmation process (commonly referred to as “transition” surgery), clearly indicating a behavior change associated with gender issues.

According to Mello et al. [58], trans people are the individuals who face the most difficulty when seeking health services. These recurring episodes of violence and refusal are motivated by both transphobia and discrimination associated with various social and cultural factors.

### 4.2. Resistance to the Use of Social Names and Pronouns

Resistance to the use of social names and pronouns was one of the most frequently emerging themes in the studies. Of the 25 studies included in this review, 14 addressed this issue [34,36,38,39,40,42,44,45,46,48,49,50,54,56].

In one study, a participant claimed that health workers only called him by his registered name, and that things only progressed, in small steps, after he changed his identity documents [48].

Other studies [45,49] show that even after requesting the use of the social name and appropriate pronoun, health workers insisted on using the registered name, disrespecting the individual’s identity [36,44]. 

According to Teixeira [59], assigning a new name is part of the identity construction process for trans people, which tends to be accompanied by physical and behavioral changes (although these changes are not a rule). Furthermore, the author states that this name carries multiple meanings and significances. Thus, not using the social name is a serious disrespect to the person [42,54,56] and is recurrently seen by the trans population as an unjustifiable lack of sensitivity [39,50].

### 4.3. Barriers to Accessing Health Services

Barriers to accessing health services are also a substantially relevant theme. In seven studies [33,37,38,41,44,50,56], the difficulties faced by the trans population in seeking health care were perceived.

In the trans population’s view, there is a lack of specialized places for their care [56]. Furthermore, many barriers stem from gender-based violence [37]. Similarly, users’ reports of not feeling comfortable discussing their gender [33,50] are common and represent a significant challenge.

When asked about the barriers, some users reported that the obstacles are created by the workers’ attitudes [41]. This allegation aligns with Rosa et al. [60], who state that a considerable portion of workers has adopted a negative stance toward trans people, creating a hostile environment and providing discriminatory and prejudiced care.

Barriers to accessing sexual and reproductive health services are also addressed in one of the studies [44]. Winter et al. [4] state that neglecting the needs of transgender women, for example, can contribute to the disproportionate risk of HIV in this group.

Lack of health insurance [33] and financial conditions [52] were also associated with difficulty accessing services, resulting in self-care and self-medication in many cases [33]. It is known that the trans population constantly deals with difficulties associated with the job market (lack of employment), which also results in low availability of resources to pay for their health [1].

It is noteworthy that only one study [40] found that the difficulty in access was associated with various factors not necessarily related to discrimination and transphobia, as they were characterized as “commonly faced” problems by users. However, the same study showed that discrimination prevents or hinders trans/transvestite people from accessing health services. This corroborates other investigations [36,49,51], as they indicate that how users are treated influences their return to consultations and the continuity of treatments. Trans people often avoid services, even in more severe health cases.

### 4.4. Discrimination and Stigma

Discrimination and stigma were also some of the most frequently mentioned themes in the studies. Out of the total analyzed, 14 addressed this type of violence [36,38,39,41,47,48,49,50,51,52,54,55,56,57].

Anticipated discrimination [56] and stigmatizing care [49] were strongly present in the studies, showing that healthcare workers often prejudge the health situations of transgender people, assuming they necessarily have sexually transmitted infections (STIs) [36,38,48,57] or that they are drug users and/or users of other illicit substances [54]. As a result, STI tests are conducted with judgmental attitudes [50], corroborating the findings of the study by Shihadeh, Pessoa, and Silva [15].

Discriminatory positions and questioning by team members [39,41,47,51,52,55] and workers from other areas [38] (such as security guards of the institutions), as well as verbal abuse and discriminatory language [38], also emerged in the analyzed articles.

Seven studies [34,38,42,49,53,56,57] documented extreme negligence and violations against transgender individuals. These situations are motivated by cultural and especially religious beliefs, revealing prejudice and religious discourses. For instance, there are reports of workers using religious books to confront the patient’s gender identity [55] and/or to reaffirm the biological factor as predominant [42]. It is known that religious discourses constitute a significant challenge, as they consider transgender people as potentially sinful [16]. These experiences, combined with other factors, lead the population to avoid returning to healthcare services [53].

Some studies also highlighted the disdain some healthcare workers have for transgender individuals. Statements like “if I had known you were trans, I wouldn’t have touched you” emerged during health appointments [57]. The excessive use of protective gear for simple procedures in these situations reveals, from the user’s perspective, considerable repulsion by the worker [49].

The absence of care or substandard care was also noted. Procedures such as suturing a wound without anesthesia [34] and focusing a medical history on gender rather than health condition [42] are present in the reports.

Due to fear of discrimination and especially of being ridiculed, many transgender people provide incomplete and/or incorrect health information to healthcare workers [55]. Moreover, due to fear of discrimination in healthcare services, transgender people have abandoned important follow-ups and treatments, worsening the phenomenon of exclusion from healthcare access [61].

It is known that transgender people prefer to seek alternative or parallel care methods [4,39]. Rocon et al. [16] report that transgender women, for example, choose to frequent “houses of worship”, where they find respect and dignity.

The way marginalized individuals are treated shapes their self-perception and how they interpret their contexts [38]. It is thus clear that discrimination is still underestimated, even though it is considered a key point for exclusion and denial of access to healthcare services [16].

### 4.5. Insensitivity of Healthcare Workers

The target population, through their statements and the authors’ analyses of the articles included in this review, mentioned episodes of insensitivity by healthcare workers, which were evident in nine publications [33,34,41,43,46,49,50,54,55].

Differentiated and discriminatory treatments, such as the absence of physical touch, lack or delay in care time [55], and changes in behavior upon learning of the patient’s transgender identity [33,34,41,43,46], were observed. Two studies [34,38] discussed the imposition of forced treatments due to hormone administration against the will of the transgender person (but with the “consent” of the family) and involuntary psychiatric care, highlighting the erasure of the individual’s desires by healthcare workers and other individuals.

For the transgender population, lack of sensitivity [33,50,54], strange looks, and disrespectful comments [48] are recurrent situations. These findings align with Winter et al.’s study [4], showing that healthcare workers are often seen as unsympathetic or hostile to the health needs of transgender people, providing inadequate care.

Almeida and Murta [62] emphasize the need to invest in the sensitization of healthcare workers, including other users of healthcare units. The authors, based on the Brazilian Ministry of Health’s Ordinance 457/2008, which regulates the transsexualizing process within the Unified Health System (the national health system), highlight the need for discrimination-free care, focusing on respect for differences and human dignity.

### 4.6. Lack of Confidence in Healthcare Services

Lack of specialized care and professional unpreparedness emerged in 11 studies [35,36,38,39,42,45,47,48,51,52,54].

The selected studies show that such situations result from healthcare workers’ lack of knowledge regarding transgender care [35,36,39,42], lack of information [38,47,48], and the absence of trans-specific and gender-affirming services [47,51,54].

Lobo et al.’s [53] study reveals that the absence of services considering the unique needs of the transgender population reduced the confidence level of the investigated transgender men and transmasculine individuals.

In one of the selected studies [49], a transgender person mentioned that they believed healthcare professionals do not have contact with trans issues during their training. This confirms studies [24,63] showing that professionals are generally unprepared to care for transgender people because they have not acquired or improved the necessary competencies to deal with this population during their training. Thus, constant training and capacity building of the entire healthcare team are necessary [39].

It is worth noting that two studies [39,56] showed that some healthcare workers’ attitudes made the transgender population feel more welcomed. These situations occurred when workers displayed, for example, flags and stickers related to the transgender population in a counseling office.

Lo and Horton [64] state that the lack of information about transgender healthcare is due to insufficient awareness and lack of acceptance of these individuals by healthcare workers. Trindade^9^ adds that institutional violence against transgender people is expressed, among other ways, by not hiring qualified and interested personnel to welcome the transgender population.

### 4.7. Technological Limitations Relating to the Binary-Focused System

Although less frequent, this section is also significantly relevant. Three studies [44,52,56] showed the challenges associated with transgender care. In this case, they report workers focused on a binary classification of sex and gender, as well as equally exclusive management systems.

Two studies reported transgender users’ complaints about limitations in health service documents and forms. According to these users, there is often no space to fill in gender identity in institutional documents [52,56].

Similarly, one study [44] shows that, in transgender users’ view, healthcare workers do not know how to deal with them because they have a limited view of the universe of sexual and gender expression possibilities. For these users, it is as if healthcare workers only see being male or being female.

It is known that discriminatory practices are based on gender and stereotypes driven by heteronormativity, besides focusing on the biomedical model [16]. Therefore, it is necessary to legitimize care based on a humanistic model centered on the patient from a holistic and comprehensive perspective [49].

Based on the results of this study, it is recommended to develop primary investigative studies, constantly train and capacitate healthcare workers, and create and/or improve clinical programs based on harm reduction, given the specificities of this population and the associated social determinants of health.

The authors of this study understand that the countries cited in the studies included in the review (Canada, Brazil, Uganda, Colombia, Turkey, USA, Spain, Nigeria, India, and Peru) have different health system organizations, and that this tends to imply different access to health services. However, no complementary analyses have been carried out to show these differences. We therefore recommend that future studies analyze and evaluate these variables.

This study included the transgender population as a whole, without considering whether they are transgender men, transgender women, or non-binary transgender people. Moreover, the specific contexts of each population, influenced by the culture and socioeconomic issues of each country and region, were not studied. The search was broad in the selected databases, allowing for a significant number of studies. For these reasons, manual searches were not conducted, nor were documents from gray literature included. Thus, future research is suggested to investigate the mentioned variables (intrinsic and extrinsic factors), broaden the scope of review, as well as conduct multicenter and field studies.

## 5. Conclusions

The analysis of the studies included in this review highlights the multiple facets of institutional violence faced by the transgender population in healthcare services. Furthermore, it is evident that forms of violence often interconnect and reinforce each other, creating a hostile environment for the transgender population in healthcare services. Therefore, the urgency of creating strategies that ensure access to dignified and respectful care for all people, regardless of their gender identity, is highlighted.

## Figures and Tables

**Table 1:** Descriptors found from automatic and manual searches.

Acronym Application		Descriptors Found
P	Transgender persons	“transgender persons”, “transgender person”, transgender, transvestisme, “sexual and gender minorities”, transvestism, “gender dysphoria”, transfeminine, “trans-Feminine”, transmasculine, “trans-Masculine”, transexual *, transsexual *, “trans people”, “trans person”, “transgender individuals”, trans
I	Violence	violence, “gender violence”, “gender-based violence”, “interpersonal violence”, “interpersonal violences”, “physical attack”
Co	Health services	“health services”, “health care services”, “healthcare services”, “health facilities”, “health care facilities”, “healthcare facilities”, “primary health care”, “primary Care”, “primary healthcare”, “secondary care”, “secondary cares”, “tertiary healthcare”, “tertiary care”, “care center”, “health services for transgender persons”, “health services accessibility”, “transgender care”, “transinclusive care”, “sexual health”, hospital *

Source: the authors.

**Table 2:** Search strategy adapted for database searches.

Base	Adapted Search Strategy
MEDLINE	(“transgender persons” [All Fields] OR “transgender person” [All Fields] OR (“transgender persons” [MeSH Terms] OR (“transgender” [All Fields] AND “persons” [All Fields]) OR “transgender persons” [All Fields] OR “transgender” [All Fields] OR “transgendered” [All Fields] OR “transgenders” [All Fields]) OR “transvestisme” [All Fields] OR “sexual and gender minorities” [All Fields] OR (“transvestic” [All Fields] OR “transvestism” [MeSH Terms] OR “transvestism” [All Fields]) OR “gender dysphoria” [All Fields] OR “transfeminine” [All Fields] OR “trans-Feminine” [All Fields] OR “transmasculine” [All Fields] OR “trans-Masculine” [All Fields] OR “transexual *” [All Fields] OR “transsexual *” [All Fields] OR “trans people” [All Fields] OR “trans person” [All Fields] OR “transgender individuals” [All Fields] OR “trans” [All Fields]) AND (“violence” [MeSH Terms] OR “violence” [All Fields] OR “violence s” [All Fields] OR “violences” [All Fields] OR “gender violence” [All Fields] OR “gender-based violence” [All Fields] OR “interpersonal violence” [All Fields] OR (“violence” [MeSH Terms] OR “violence” [All Fields] OR (“interpersonal” [All Fields] AND “violences” [All Fields])) OR “physical attack” [All Fields]) AND (“health services” [All Fields] OR “health care services” [All Fields] OR “healthcare services” [All Fields] OR “health facilities” [All Fields] OR “health care facilities” [All Fields] OR “healthcare facilities” [All Fields] OR “primary health care” [All Fields] OR “primary Care” [All Fields] OR “primary healthcare” [All Fields] OR “secondary care” [All Fields] OR “secondary cares” [All Fields] OR “tertiary healthcare” [All Fields] OR “tertiary care” [All Fields] OR “care center” [All Fields] OR “health services for transgender persons” [All Fields] OR “health services accessibility” [All Fields] OR “transgender care” [All Fields] OR “transinclusive care” [All Fields] OR “sexual health” [All Fields] OR “hospital*” [All Fields])
Scopus	TITLE-ABS-KEY (“transgender persons” OR “transgender person” OR transgender OR transvestisme OR “sexual and gender minorities” OR transvestism OR “gender dysphoria” OR transfeminine OR “trans-Feminine” OR transmasculine OR “trans-Masculine” OR transexual * OR transsexual * OR “trans people” OR “trans person” OR “transgender individuals” OR trans) AND TITLE-ABS-KEY (violence OR “gender violence” OR “gender-based violence” OR “interpersonal violence” OR “interpersonal violences” OR “physical attack”) AND TITLE-ABS-KEY (“health services” OR “health care services” OR “healthcare services” OR “health facilities” OR “health care facilities” OR “healthcare facilities” OR “primary health care” OR “primary Care” OR “primary healthcare” OR “secondary care” OR “secondary cares” OR “tertiary healthcare” OR “tertiary care” OR “care center” OR “health services for transgender persons” OR “health services accessibility” OR “transgender care” OR “transinclusive care” OR “sexual health” OR hospital *)
EMBASE	#1 ‘transgender persons’/exp OR ‘transgender persons’ OR ‘transgender person’/exp OR ‘transgender person’ OR ‘transgender’/exp OR transgender OR transvestisme OR ‘sexual and gender minorities’/exp OR ‘sexual and gender minorities’ OR ‘transvestism’/exp OR transvestism OR ‘gender dysphoria’/exp OR ‘gender dysphoria’ OR transfeminine OR ‘trans-feminine’ OR transmasculine OR ‘trans-masculine’ OR transexual * OR transsexual * OR ‘trans people’/exp OR ‘trans people’ OR ‘trans person’/exp OR ‘trans person’ OR ‘transgender individuals’ OR trans #2 ‘violence’/exp OR violence OR ‘gender violence’/exp OR ‘gender violence’ OR ‘gender-based violence’/exp OR ‘gender-based violence’ OR ‘interpersonal violence’/exp OR ‘interpersonal violence’ OR ‘interpersonal violences’ OR ‘physical attack’ #3 ‘health services’/exp OR ‘health services’ OR ‘health care services’ OR ‘healthcare services’ OR ‘health facilities’/exp OR ‘health facilities’ OR ‘health care facilities’ OR ‘healthcare facilities’ OR ‘primary health care’/exp OR ‘primary health care’ OR ‘primary care’/exp OR ‘primary care’ OR ‘primary healthcare’/exp OR ‘primary healthcare’OR ‘secondary care’/exp OR ‘secondary care’ OR ‘secondary cares’ OR ‘tertiary healthcare’/exp OR ‘tertiary healthcare’ OR ‘tertiary care’/exp OR ‘tertiary care’ OR ‘care center’ OR ‘health services for transgender persons’/exp OR ‘health services for transgender persons’ OR ‘health services accessibility’/exp OR ‘health services accessibility’ OR ‘transgender care’ OR ‘transinclusive care’ OR ‘sexual health’/exp OR ‘sexual health’ OR hospital* #4 #1AND #2 AND #3 #5 #4AND [embase]/lim
Web of Science	“transgender persons” OR “transgender person” OR transgender OR transvestisme OR “sexual and gender minorities” OR transvestism OR “gender dysphoria” OR transfeminine OR “trans-Feminine” OR transmasculine OR “trans-Masculine” OR transexual * OR transsexual* OR “trans people” OR “trans person” OR “transgender individuals” OR trans (Topic) and violence OR “gender violence” OR “gender-based violence” OR “interpersonal violence” OR “interpersonal violences” OR “physical attack” (Topic) and “health services” OR “health care services” OR “healthcare services” OR “health facilities” OR “health care facilities” OR “healthcare facilities” OR “primary health care” OR “primary Care” OR “primary healthcare” OR “secondary care” OR “secondary cares” OR “tertiary healthcare” OR “tertiary care” OR “care center” OR “health services for transgender persons” OR “health services accessibility” OR “transgender care” OR “transinclusive care” OR “sexual health” OR hospital* (Topic)
CINAHL	(“transgender persons” OR “transgender person” OR transgender OR transvestisme OR “sexual and gender minorities” OR transvestism OR “gender dysphoria” OR transfeminine OR “trans-Feminine” OR transmasculine OR “trans-Masculine” OR transexual* OR transsexual* OR “trans people” OR “trans person” OR “transgender individuals” OR trans) AND (violence OR “gender violence” OR “gender-based violence” OR “interpersonal violence” OR “interpersonal violences” OR “physical attack”) AND (“health services” OR “health care services” OR “healthcare services” OR “health facilities” OR “health care facilities” OR “healthcare facilities” OR “primary health care” OR “primary Care” OR “primary healthcare” OR “secondary care” OR “secondary cares” OR “tertiary healthcare” OR “tertiary care” OR “care center” OR “health services for transgender persons” OR “health services accessibility” OR “transgender care” OR “transinclusive care” OR “sexual health” OR hospital*)
LILACS	(“transgender persons” OR “transgender person” OR transgender OR transvestisme OR “sexual and gender minorities” OR transvestism OR “gender dysphoria” OR transfeminine OR “trans-Feminine” OR transmasculine OR “trans-Masculine” OR transexual* OR transsexual* OR “trans people” OR “trans person” OR “transgender individuals” OR trans OR “pessoas transgênero” OR “pessoa transgênero” OR transgênero OR travestismo OR “minorias sexuais e de gênero” OR “disforia de gênero” OR transfeminino OR “trans-Feminino” OR transmasculino OR “trans-Masculino” OR transexual* OR “pessoas trans” OR “pessoa trans” OR “indivíduos trans” OR “personas transgénero” OR “persona transgénero” OR transgénero OR travestismo OR “minorías sexuales y de género” OR “disforia de género” OR transfemenino OR “persona trans” OR “individuos transgénero”) AND (violence OR “gender violence” OR “gender-based violence” OR “interpersonal violence” OR “interpersonal violences” OR “physical attack” OR violência OR “violência de gênero” OR “violência interpessoal” OR “violências interpessoais” OR “ataque físico” OR violencia OR “violencia de género” OR “violencia interpersonal” OR “violencias interpersonales”) AND (“health services” OR “health care services” OR “healthcare services” OR “health facilities” OR “health care facilities” OR “healthcare facilities” OR “primary health care” OR “primary Care” OR “primary healthcare” OR “secondary care” OR “secondary cares” OR “tertiary healthcare” OR “tertiary care” OR “care center” OR “health services for transgender persons” OR “health services accessibility” OR “transgender care” OR “transinclusive care” OR “sexual health” OR hospital* OR “serviços de saúde” OR “estabelecimentos de saúde” OR “cuidados de saúde primários” OR “cuidados secundários”or “cuidados de saúde terciários” OR “cuidados terciários” OR “centro de cuidados” OR “serviços de saúde para pessoas trans” OR “acessibilidade aos serviços de saúde” OR “atendimento a transgêneros” OR “atendimento transinclusivo” OR “atendimento sexual saúde” OR “servicios de salud” OR “servicios de atención médica” OR “centros de salud” OR “centros de atención médica” OR “atención primaria de salud” OR “atención primaria” OR “ atención secundaria” OR “atención médica terciaria” OR “atención terciaria” OR “centro de atención” OR “servicios de salud para personas transgénero” OR “accesibilidad a servicios de salud” OR “atención a personas transgénero” OR “atención transinclusiva” OR “atención sexual salud”) AND (db:(“LILACS”))

Legend: We searched the EMBASE database for terms separately. Each symbol # represents a search. The last two lines are the combination of searches / We chose the symbol * because in certain words it represents the word stem. Source: the authors.

**Table 3:** Information and synthesis of the main results of studies selected for systematic review.

ID	Author/Year of Publication/Journal/Country	Study Objective	Place of Study/Participants	Data Collection/Data Analysis	Main Findings
A1 [33]	Xavier et al./2013/*International Journal of Transgenderism*/USA	Identify the factors associated with greater risk of HIV infection and the principal social determinants of health status among transgender people in Virginia.	Virginia, USA/32 trans women and 15 trans men participated of the study	Seven focus groups and one individual interview were conducted/Initial descriptive coding was performed separately on the transcriptions line by line using NVivo 2.0 software	Victimization associated with social stigmatization played a dominant role in participants’ lives, manifested by discrimination; violence; and health care provider insensitivity, hostility, and ignorance of transgender health. Access to transgender-related medical services that would allow for participants to pass in their chosen genders was their highest medical priority. Faced with barriers to access, hormonal self-medication was common, and silicone injections were reported by both MtF and FtM participants. Due to economic vulnerability, sex work was reported as a source of income by both MtFs and FtMs. MtFs expressed concern over confidentiality of HIV testing and additional discrimination if testing positive. FtMs expressed difficulty accessing gynecological care due to their masculine gender identities and expressions.
A2 [34]	Kosenko et al./2013/*Medical Care*/USA	To explore the negative experiences of transgender individuals in health care settings.	USA/152 self-identified transgender adults from 40 different states and 2 foreign countries participated.	Data collected in 2010 as part of an IRB-approved needs assessment of transgender adults. Questionnaire completed online and through mailings to LGBTQ organizations/Data were collected and analyzed according to Morse and Field’s conventional qualitative content analytic approach.	Participants reported mistreatment in health care contexts due to gender identity or presentation. Problematic provider behaviors included gender insensitivity, displays of discomfort, denial of services, substandard care, verbal abuse, and forced care.
A3 [35]	Lyons et al./2015/*Substance Abuse Treatment, Prevention and Policy*/Canada	Qualitatively investigate the treatment experiences of transgender individuals using illicit drugs in a residential dependency setting in a Canadian environment.	Vancouver, Canada/34 transgender people participated.	In-depth semi-structured interviews were conducted/The interviews were transcribed verbatim and imported into Atlas.ti software (version 8.3.0.)/Theoretical thematic analysis and a participatory analysis approach were applied	Three themes emerged from the data characterizing individuals’ experiences in treatment settings: (1) stigma enacted through social rejection and violence, (2) felt transphobia and stigma, and (3) “trans-friendly” and inclusive treatment. Participants who reported feeling and experiencing stigma, including violence, prematurely left the treatment after experiencing isolation and conflicts. In contrast, participants who felt included and respected in the treatment settings reported positive treatment experiences.
A4 [36]	Souza et al./2015/*Cadernos de Saúde Pública*/Brazil	The article discusses the violence experienced by transvestites (in the family, school, police precincts, and health services), specifically seeking to understand how such violence relates to their experiences with health services and how the latter respond.	Rio Grande do Sul, Brazil/49 transvestites participated	The authors conducted an ethnographic research with transgender persons in Santa Maria, Rio Grande do Sul State, Brazil, in 2012, using participant observation, semi-structured interviews, and following their everyday lives/The observations and narratives that emerged from the field were transcribed, coded, thematically categorized, and compared with theoretical concepts from gender studies, violence studies, and the field of public health.	The various forms of violence experienced by transgender women throughout their lives directly interfere with their health conditions. Besides distancing them from their nuclear families and kinship relations, thereby removing material support and emotional connections, it also pushes them away from schools and health services, which, as we have seen, replicate violence, contributing to their suffering. The physical and symbolic violence and the resulting suffering were constants that participants had to deal with in their daily practices and routines.
A5 [37]	Lyons et al./2016/*LGBT Health*/Canada	Investigate the experiences of trans women and Two-Spirit people in accessing women-specific services in the Downtown Eastside of Vancouver.	Vancouver, Canada/32 trans women and two-spirit individuals participated.	In-depth semi-structured interviews were conducted/The interview data were analyzed using a participatory and inductive analysis approach.	Participants generally managed to access women-specific services in the neighborhood. However, there were reports of discrimination related to gender identity, discrimination based on gender expression (e.g., the requirement for a feminine gender expression), and lack of staff intervention in the harassment by other service users.
A6 [38]	Ritterbusch; Salazar; Correa/2018/*Global Public Health*/Colombia	Present the stigma-related barriers to healthcare experienced by trans women and their experiences of multi-level violence within the healthcare system.	Colombia/28 transgender women participated	Semi-structured interviews were conducted within a participatory action research framework/The interviews were coded using a grounded theory approach.	Trans women experience violence at various levels, from intimate bodily violence to the formulation of high-level public health policies.
A7 [39]	Samuels et al./2018/*Annals of Emergency Medicine*/USA	To understand the experiences of transgender adults with at least one emergency department visit in the past 5 years and identify barriers and suggestions for improving emergency care for this population.	Oregon, USA/32 transgender adults participated in 4 focus groups, with most participants being white, preferring male pronouns, and identifying as female to male, transgender, or male.	Focus group discussions facilitated by the study principal investigator and another research team member, lasting approximately 2 h and recorded digitally/Transcripts were professionally transcribed, identified, and analyzed using qualitative data management software (NVivo).	Experiences of harassment and assault were common among participants, with the majority experiencing verbal harassment, physical assault, and sexual assault. While most had a primary care provider and insurance, only a small percentage had insurance plans covering sex affirmation or reassignment health services. Nearly half reported avoiding the emergency department when in need of acute care.
A8 [40]	Monteiro; Brigeiro/2019/*Cadernos de Saúde Pública*/Brazil	Analyzing the access experience of trans/transvestite women to the healthcare service.	Baixada Fluminense, Rio de Janeiro, Brazil/9 trans/transvestite women participated.	Individual and semi-structured interviews were conducted with trans women/transvestites from lower-income backgrounds in Baixada Fluminense, Rio de Janeiro.	Comparing to past experiences of aggression, narratives from trans women/transvestites highlight social advancements. They report that professionals generally do not discriminate based on their condition, though there is resistance to using their preferred name.
A9 [41]	King et al./2019/*Culture, Health & Sexuality*/Uganda	Exploring HIV and gender-related contexts among transgender women.	Kampala, Uganda/45 trans/transvestite women participated	Computer-assisted self-interviews, in-person qualitative interviews, and HIV and CD4 blood tests were conducted. Recruitment occurred through snowball sampling/Quantitative interviews utilized Questionnaire Design Studio (QDS v2.5). Trained qualitative social scientists coded and analyzed transcripts using NVivo Version 11 for data management, employing content analysis.	Nearly all interviewees reported frequently engaging in sex work, primarily due to lack of employment. HIV-related themes included limited access to non-stigmatizing health services, inconsistent condom use, inaccurate perceptions of risk for themselves and their partners, alcohol use, receptive anal sex with men, multiple sexual partners, frequent self-stigma, and enacted violence.
A10 [42]	Goldenberg et al./2019/*The Counseling Psychologist*/USA	Understanding the mental health experiences and healthcare of participants, including factors related to gender misalignment and less affirmative treatment by providers.	USA/506 transgender undergraduate and graduate students participated in the study.	It is a mixed methods study involving an online questionnaire with both open-ended and closed-ended questions. Thematic analysis was employed in the qualitative phase.	The prominent characteristics of negative interactions included invalidation, avoidance, or excessive focus on participants’ non-binary identities. Non-binary students reported more gender misidentifications by therapists and healthcare professionals, and less trans-affirmative care from healthcare providers, compared to binary students.
A11 [43]	Dutta; Khan; Lorway/2019/*Culture, Health & Sexuality*/India	Describing the effects of overlapping forms of structural violence around education, livelihood, family life, and attempts to access social and healthcare services.	Karnataka, India/3 transgender people participated.	Ethnographic research was conducted using thematic interpretative analysis and inductive reasoning.	The findings indicate how social inequalities contribute to the development of transgender identities along the journey to becoming a “jogappa”. They emphasize the evolving needs of transgender individuals in India, which are rooted in moral narratives of religiosity, urging policymakers to take these diverse needs into account.
A12 [44]	Calderón-Jaramillo et al./2020/*International Journal for Equality in Health*/Colombia	Identifying the primary sexual and reproductive health needs of individuals living a trans life; generating new evidence to guide the adaptation of sexual and reproductive health services centered on the needs, identities, and circumstances of trans people.	Barranquilla, Bogota, Cali and Medellín, Colombia/13 transgender people participated.	Focus group discussions and in-depth interviews were conducted for a qualitative study from a constructivist perspective. NVivo software was utilized for data coding and analysis.	Among the main barriers encountered were healthcare costs, lack of insurance, stigma, discrimination, and abuse by healthcare professionals. Some of the most notable sexual and reproductive health needs included trans-specific services, such as sensitive assistance for the transition process, endocrinology consultations, and sexual affirmation surgeries.
A13 [45]	Silva et al./2020/*Revista Brasileira de Enfermagem*/Brazil	Analyzing the health vulnerability of young transgender women living with HIV/AIDS.	Recife, Pernambuco, Brazil/6 transgender women participated.	Semi-structured interviews were conducted for a qualitative, descriptive, and exploratory study grounded in the theoretical framework of Social Representation and vulnerability concept. Individual interviews were analyzed, recorded, and fully transcribed using IRaMuTeQ software (version 0.7) for Similarity Analysis.	Young transgender women living with HIV/AIDS experience health vulnerability associated with lack of knowledge and difficulties in practicing self-care. There were representations of social abjection and unpreparedness among primary healthcare teams in providing qualified assistance for effective and humane care.
A14 [46]	Lacombe-Duncan; Olawale/2020/*Journal of Interpersonal Violence*/Canada	Understanding the context, types, and consequences of violence experienced by transgender women living with HIV across their lives, from intersectional and social–ecological perspectives.	Canada/Eleven transgender women living with HIV participated in the study.	Semi-structured individual interviews were conducted. Structural analysis was used to identify key themes, patterns within themes across participants, and patterns across themes among participants.	The findings revealed that transgender women living with HIV experience specific contexts of violence shaped at the intersection of stigma based on gender identity, gender expression, HIV status, and other identities/experiences. Once living with HIV, transgender women were subjected to discursive violence from healthcare professionals. These multiple forms of violence have serious consequences for the social, mental, and physical well-being of transgender women living with HIV.
A15 [47]	Reisner et al./2021/*PLoS ONE*/Peru	The user aims to increase visibility, document, and understand the health needs and contexts shaping the health and well-being of transgender men in Lima, Peru, to inform responsive public health efforts.	Lima, Peru/46 transgender men participated	User conducted 4 focus groups and 10 individual interviews. Analysis was conducted using an immersion crystallization approach to identify themes.	Transgender men reported lack of awareness and information among medical providers, avoidance of healthcare due to discrimination and mistreatment, absence of public services for medical gender affirmation (hormones, surgeries), and unmet mental health needs. They described health as multidimensional and influenced by social, economic, and legal contexts, including family, school, employment, legal identity recognition, discrimination in public spaces, and peer support. Violence, stigma, and intersecting forms of oppression were described as limiting social and legal recognition of transgender identity, a central dimension of health. Peer support, often in online spaces, was described as important for resilience and well-being.
A16 [48]	Oliveira et al./2022/*Revista Brasileira de Enfermagem*/Brazil	Understanding the meanings of being a transgender woman or transvestite in the healthcare provided by professionals of the Unified Health System.	Minas Gerais, Brazil/10 transgender women or transvestites residents and users of the Unified Health System (Sistema Único de Saúde) participated.	Interviews were conducted/Heideggerian Phenomenology was used as the theoretical, methodological, and philosophical foundation	Transgender women and transvestites often conform to socially constructed and accepted feminine patterns, frequently seeking hormone therapy. They sometimes resort to self-medication when facing difficulties obtaining prescriptions. Acceptance and use of their chosen name by healthcare professionals promote their recognition. These individuals experience daily prejudice, not only from professionals but also from assumptions made by other service users.
A17 [49]	Santander-Morillas et al./2022/*PLoS ONE*/Spain	Describing the experiences of transgender individuals regarding the healthcare they received in primary and hospital services in Barcelona from 2017 to 2019.	Barcelona, Espanha/16 transgender people participated	Semi-structured interviews were conducted based on a descriptive phenomenological approach. Data were analyzed descriptively and thematically following the method proposed by Colaizzi, aided by Atlas.ti 8 software.	The experiences of transgender care were divided into three categories: overcoming obstacles, training consultations, and coping strategies. Participants identified negative experiences and challenges with the healthcare system due to the lack of competence among healthcare professionals. Discriminatory, authoritarian, and paternalistic behaviors continue to exist, hindering therapeutic relationships, care, and access to health services.
A18 [50]	Tun et al./2022/*J Internacional AIDS Society*/Nigeria	Evaluate how stigma influences HIV services for transgender individuals in Lagos, Nigeria.	Lagos, Nigeria/25 transgender women and 13 transgender men participated.	In-depth interviews and focus group discussions were conducted. Thematic content analysis was used to analyze the data, utilizing NVivo 12 software.	The disclosure of gender identity is challenging due to anticipated stigma experienced by transgender individuals and fear of legal repercussions. Fear of being reported to authorities was a major barrier to disclosing to providers in non-affiliated transgender-inclusive clinic facilities. Participants reported a lack of sensitivity among providers regarding gender identity, with instances of confusion between transgender men and lesbian women, and transgender women with gay men or men who have sex with men, the latter being more common. Transgender participants also expressed feeling disrespected when providers were not sensitive to their preferred pronouns. HIV services that are not transgender-inclusive and affirming can reinforce stigma.
A19 [51]	Ssekamatte et al./2022/*International Journal for Equity in Health*/Uganda	Exploring sources of gender-based violence support services and the challenges faced by transgender women in seeking help.	Kampala, Uganda/60 transgender women and 10 key informants participated.	Deep interviews and a focus group discussion guide were utilized. Recruitment was through snowball sampling. Data were transcribed verbatim and analyzed following a thematic structure informed by the socioecological model.	Lack of recognition of transgender identity; long distances to health facilities; discrimination by healthcare professionals and civil society organization staff; inadequate questioning of transgender identity by police and healthcare providers; and the lack of transgender-competent healthcare professionals and legal personnel hindered seeking help after exposure to gender-based violence.
A20 [52]	Sherman et al./2022/*PLoS ONE*/EUA	To explore the help-seeking process post-exposure to violence among Black transgender women and the association between polyvictimization, barriers to healthcare, and mental health outcomes.	Baltimore e Washington, United States of America/Nineteen transgender women participated in the qualitative stage of the study.	Semi-structured interviews were conducted. Analyses included thematic content analysis, bivariate analysis, joint display, and multivariate linear regression analysis examining mediation and moderation.	The study found that barriers to accessing and engaging in healthcare, polyvictimization, and mental health symptom severity were interconnected among Black transgender women. The research highlighted the importance of addressing these barriers to improve overall well-being in this population.
A21 [53]	Lobo et al./2023/*Revista Brasileira de Enfermagem*/Brazil	Analyzing the repercussions of transphobia on the health of transgender men and transmasculine individuals.	Bahia, Brazil/Thirty-eight individuals participated, including 35 transgender men and three transmasculine individuals.	In-depth interviews were conducted. The Collective Subject Discourse technique was employed, and interpretation was based on the theoretical concept of transphobia.	Transphobia has brought intra and interpersonal repercussions in the lives and health of transgender men and transmasculine individuals who access healthcare services. Experiences of violence in private spaces, strained family ties, discrimination in educational settings, limitations in professional opportunities/employment, barriers in self-care and access to healthcare services, development of strategies to protect transgender identity, and consequences of transphobia on psychosocial health were found.
A22 [54]	Jesus et al./2023/*Interface (Botucatu)*/Brazil	Understanding how transgender women have been treated in institutions of the Unified Health System (Sistema Único de Saúde).	Minas Gerais, Brazil/Four transgender women participated.	Focus group interviews were conducted, and thematic content analysis was performed.	The participants mentioned not having ties with Primary Care and seeking care at the outpatient clinic linked to the teaching hospital and emergency services. Instances of institutional violence, negligence, and prejudice permeate their experiences.
A23 [55]	Muyanga et al./2023/*BMC Women’s Health*/Uganda	Exploring how gender-based violence affects the uptake and utilization of HIV prevention, treatment, and care services among transgender women in the greater Kampala metropolitan area, Uganda.	Kampala, Uganda/60 transgender women participated.	Twenty in-depth interviews, six focus group discussions, and ten interviews with key informants were conducted. Data were analyzed using a thematic content analysis framework. Verbatim transcription of data was performed, and NVivo 12 was used for coding.	Physical and emotional violence at the community level has led to fear among transgender women traveling to healthcare facilities. Emotional violence experienced by transgender women in healthcare settings has resulted in limited use of pre-exposure prophylaxis and HIV testing services, denial of healthcare services, and delays in receiving appropriate care. Fear of emotional violence has also made it difficult for transgender women to approach healthcare professionals. Fear of physical violence, such as being assaulted while in healthcare settings, has caused transgender women to avoid healthcare facilities.
A24 [56]	Burchell et al./2023/*Culture, Health & Sexuality*/Canada	Understanding the barriers to healthcare among non-binary individuals living in a medium-sized urban/rural region of Canada.	Waterloo, Ontario, Canada/12 non-binary individuals participated.	Semi-structured interviews were conducted. The theoretical framework of this study was based on interpretative phenomenology. Transcribed interviews were thematically analyzed using NVivo.	Three overarching themes were developed: erasure, barriers to accessing healthcare, and assessing whether (or not) to seek care. Subthemes included institutional erasure, informational erasure, general health barriers, medical transition barriers to healthcare, anticipated discrimination, and safety assessment.
A25 [57]	Atuk/2024/*Social Science & Medicine* /Turkey	Examining the medical experiences of HIV-positive trans women who engage in sex work and the harmful violence they encounter at the hands of healthcare professionals.	Turkey/10 women participated	As part of a broader research initiative, this article integrates multiple data sources, including public archives, medical records, newspaper articles, official government reports, and 45 in-depth interviews with healthcare providers/When it came to the inclusion of the study, it focused on in-dept interviews carried out with trans woman/thematic analysis was applied using ethnographically informed interpretive frameworks.	Trans women are treated by healthcare professionals as if they were always infectious. Trans communities are slowly weakened by the denial of healthcare services.

Source: adapted from Aromataris et al. [32].

**Table 4:** Methodological quality of articles included in the systematic review.

ID	Is There Congruence between the Stated Philosophical Perspective and the Research Methodology?	Is There Congruence between the Research Methodology and the Research Question or Objectives?	Is There Congruence between the Research Methodology and the Methods Used to Collect the Data?	Is There Congruence between the Research Methodology and the Representation and Analysis of Data?	Is There Congruence between the Research Methodology and the Interpretation of Results?	Is There a Statement Locating the Researcher Culturally or Theoretically?	Is the Researcher’s Influence on Research and Vice Versa Addressed?	Are Participants and Their Voices Adequately Represented?	Is the Research Ethical According to Current Criteria or, Recent Studies, for Is There Evidence of Ethical Approval by an Appropriate Body?	Do the Conclusions Drawn in the Research Report Stem from Data Analysis or Interpretation?
A1 [33]	Y	Y	Y	Y	Y	Y	U	Y	Y	Y
A2 [34]	U	Y	Y	Y	Y	U	U	Y	Y	Y
A3 [35]	Y	Y	Y	Y	Y	Y	Y	Y	Y	Y
A4 [36]	U	Y	Y	Y	Y	U	U	Y	Y	Y
A5 [37]	Y	Y	Y	Y	Y	N	U	Y	Y	Y
A6 [38]	Y	Y	Y	Y	Y	Y	U	Y	Y	Y
A7 [39]	Y	Y	Y	Y	Y	U	U	Y	Y	Y
A8 [40]	U	Y	Y	Y	Y	Y	U	Y	Y	Y
A9 [41]	U	Y	Y	Y	Y	Y	U	Y	Y	Y
A10 [42]	Y	Y	Y	Y	Y	Y	Y	Y	Y	Y
A11 [43]	Y	Y	Y	Y	Y	Y	Y	Y	Y	Y
A12 [44]	Y	Y	Y	Y	Y	Y	Y	Y	Y	Y
A13 [45]	Y	Y	Y	Y	Y	Y	Y	Y	Y	Y
A14 [46]	Y	Y	Y	Y	Y	Y	Y	Y	Y	Y
A15 [47]	U	Y	Y	Y	Y	U	U	Y	Y	Y
A16 [48]	U	Y	Y	Y	Y	U	U	Y	Y	Y
A17 [49]	U	Y	Y	Y	Y	Y	U	Y	Y	Y
A18 [50]	Y	Y	Y	Y	Y	Y	U	Y	Y	Y
A19 [51]	Y	Y	Y	Y	Y	U	U	Y	Y	Y
A20 [52]	Y	Y	Y	Y	Y	Y	U	Y	Y	Y
A21 [53]	Y	Y	Y	Y	Y	Y	U	Y	Y	Y
A22 [54]	Y	Y	Y	Y	Y	U	U	Y	Y	Y
A23 [55]	U	Y	Y	Y	Y	U	U	Y	Y	Y
A24 [56]	Y	Y	Y	Y	Y	Y	Y	Y	Y	Y
A25 [57]	U	Y	Y	Y	Y	Y	Y	Y	U	Y

Legend: Y (yes); N (No); U (Unclear); source: based on Aromataris et al. [32].

## Data Availability

Not applicable.
